# Supplementary material for: Uncovering Novel Pre-Treatment Molecular Biomarkers for Anti-TNF Therapeutic Response in Patients with Crohn’s Disease
Source: J Funct Biomater. 2022 Mar 30;13(2):36. doi: 10.3390/jfb13020036 (PMC9036297; doi:10.3390/jfb13020036)
Supplement: Supplementary file 1 [file jfb-13-00036-s001.zip › jfb-1650263-supplementary.pdf]

Supplementary

Table S1. Detailed information of the GEO datasets.

	GSE16879	GSE42296
Platform	Affymetrix Human Genome U133 Plus 2.0 Array	Affymetrix Human Gene 1.0 ST Array
Source	Ileo-colonic tissue	PBMC
Anti-TNF agent	Infliximab	Infliximab
Drug-response definition	At 4 to 6 weeks, ≥3-point decrease on the histological score compared with baseline	At 6 weeks, >100-point decrease in CDAI compared with baseline

PBMC, peripheral blood mononuclear cell; Anti-TNF, anti-tumor necrosis factor; CDAI, Crohn's disease activity index.

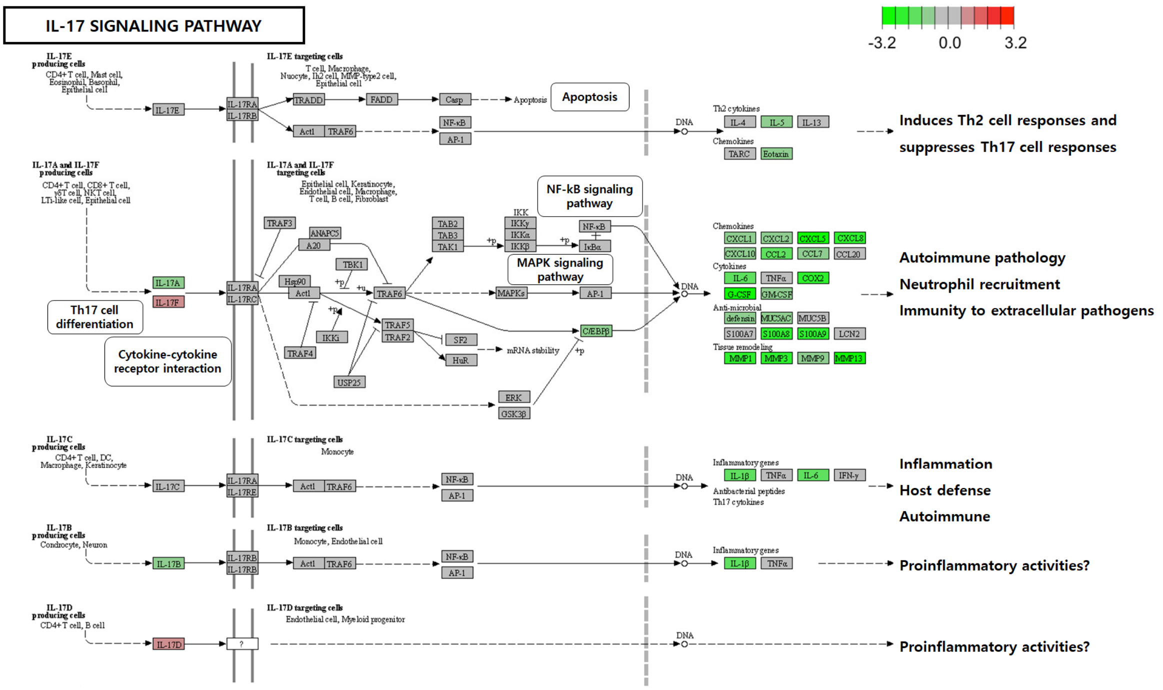

Figure S1. Expression changes of target genes associated with anti-TNF therapy resistance in Crohn's disease in the IL-17 signalling pathway are mapped by colours. Red colour—statistically significant increase in expression, green colour—statistically significant decrease in expression, and grey colour—expression statistically insignificant.

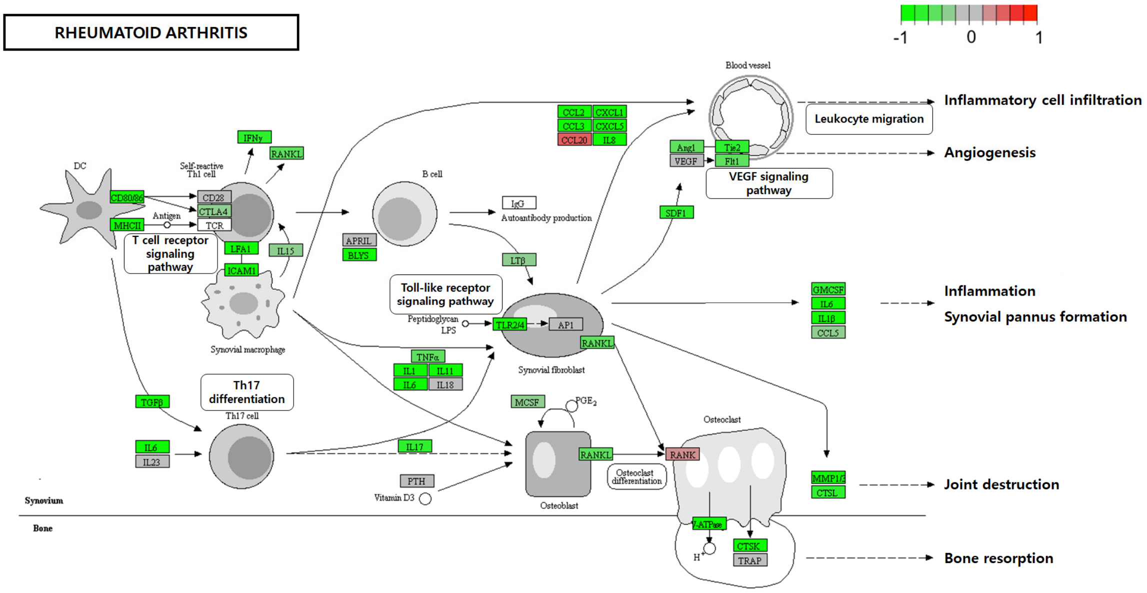

Figure S2. Expression changes of target genes associated with anti-TNF therapy resistance in Crohn's disease in the rheumatoid arthritis pathway are mapped by colours. Red colour—statistically significant increase in expression, green colour—statistically significant decrease in expression, grey colour—expression statistically insignificant.

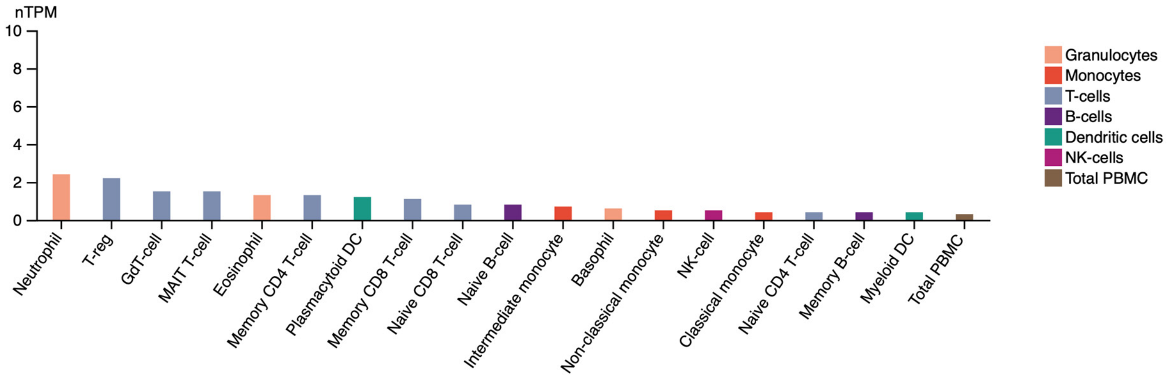

Figure S3. Interaction between the *KAT2B* gene and immune cells in normal human tissues analysed using the Human Protein Atlas database. nTPM, normalized units of transcript per million.
